# Factors associated with severe pneumonia in adults hospitalised with community-acquired pneumonia in Mongolia

**DOI:** 10.1186/s12889-025-26109-2

**Published:** 2026-01-03

**Authors:** Amy Simkiss, Munkhchuluun Ulziibayar, Purevsuren Batsaikhan, Bujinlkham Suuri, Dashtseren Luvsantseren, Dorj Narangerel, Bilegtsaikhan Tsolmon, Eileen M. Dunne, Bradford D. Gessner, E. Kim Mulholland, Tuya Mungun, Claire von Mollendorf

**Affiliations:** 1 Department of Paediatrics, University of Melbourne, Melbourne, Australia; 2 National Center for Communicable Diseases, Ministry of Health, Ulaanbaatar, Mongolia; 3 Ministry of Health, Ulaanbaatar, Mongolia; 4 Mongolian National University of Medical Sciences, Ulaanbaatar, Mongolia; 5 Pfizer Vaccines, Collegeville, USA; 6 Infection, Immunity and Global Health, Murdoch Children’s Research Institute, Melbourne, Australia; 7 London School of Hygiene and Tropical Medicine, London, UK

**Keywords:** Community acquired pneumonia, Severe pneumonia, Mongolia, Adults

## Abstract

**Background:**

Limited research has focused on risk factors for community acquired pneumonia (CAP) in adults in low-middle income countries (LMICs). Mongolia has high rates of CAP hospitalisations with extreme winter temperatures and significant air pollution. We aimed to examine risk factors for severe CAP among hospitalised adults aged ≥ 18 years in Mongolia.

**Methods:**

Adults hospitalised with clinical CAP were enrolled over three years (2019–2022) into a prospective CAP surveillance program in four district hospitals in the capital city, Ulaanbaatar. Participants had clinical information and risk factors collected using a case report form. Nasopharyngeal swabs were collected and tested for *Streptococcus pneumoniae* (the pneumococcus), influenza and respiratory syncytial virus, while collected urine was tested for the pneumococcus. From 2020 only patients with a negative SARS-CoV-2 test were enrolled in the study. Severe CAP was defined as clinical CAP with intensive care unit admission or ≥ 2 severity signs (confusion, hypotension, tachypnoea or hypoxaemia). Multivariable logistic regression was used to derive adjusted risk factor estimates.

**Results:**

Overall, 3178 participants met the CAP study case definition and 10.1% had severe CAP. Pneumococcal carriage (aOR:2.67; 95% CI:1.88–3.79; *p* < 0.001), pneumococcal serotype-specific urine antigen detection positivity (aOR:1.69; 95% CI:1.11–2.57; *p* = 0.01), hospital admission within the last year (aOR:1.73; 95% CI:1.26–2.37; *p* = 0.001), underlying medical conditions (aOR:1.96; 95% CI:1.39–2.76; *p* < 0.001), cigarette smoking (aOR:1.53; 95% CI:1.08–2.16; *p* = 0.01) and alcohol intake (aOR:2.19; 95% CI:1.49–3.22; *p* < 0.001), were identified as risk factors for severe CAP on multivariable analysis.

**Conclusion:**

We identified key risk factors for severe CAP in hospitalised adults in urban Mongolia. If resources are limited, this data could be used to prioritise groups for vaccinations, alongside public health policies to decrease tobacco smoking and alcohol consumption.

**Supplementary Information:**

The online version contains supplementary material available at 10.1186/s12889-025-26109-2.

## Background

Community acquired pneumonia (CAP) remains a major cause of morbidity and mortality in adults worldwide [1, 2]. *Streptococcus pneumoniae* (the pneumococcus) is the leading cause of CAP in adults accounting for around 27% of cases as a conservative estimate [1–3]. The Global Burden of Disease study estimated that in 2021 more than 75 million people aged over 15 years experienced pneumococcal disease while approximately 350,000 people in this age group died of pneumococcal disease [4]. In 2019, prior to the COVID-19 pandemic, influenza was the third most common causative agent of lower respiratory infections and deaths [4]. A 2021 global meta-analysis estimated the influenza virus was responsible for more than 5 million hospitalisations in adults with lower respiratory infections [5].

Research on adult CAP burden and risk factors from low-middle income countries (LMICs) is limited. This data gap reduces the ability to develop the most efficient public health programs for these settings due to fundamental differences in social determinants of health in LMICs compared with high-income countries (HICs) [1, 6]. Risk factors described for adult CAP from research in HICs include previous CAP diagnosis in the past two years, underlying medical conditions, older age (≥ 60 years), and lifestyle factors such as cigarette smoking and increased alcohol intake [1, 7–12]. In LMICs the most frequently described risk factors include older age (≥ 60 years), smoking, household crowding and environmental hazards such as indoor and outdoor pollution and exposure to environmental substances [1, 12, 13]. In HICs some data on severe CAP suggests high alcohol intake and underlying medical conditions worsen outcomes for patients [14, 15]. Pneumococcal carriage or disease and bacterial and viral coinfection are also associated with more severe respiratory disease and increased complications [16–21].

Mongolia is a landlocked country that experiences cold winters and considerable levels of air pollution, especially in the capital city, Ulaanbaatar, which is home to almost half of the total population [22, 23]. With an aging population and prevalent tobacco and alcohol use, there is a high burden of respiratory disease with CAP accounting for around half of respiratory hospital admissions across all age groups in 2018 [24–26]. Annual influenza vaccination campaigns target high risk groups to try and reduce the burden of respiratory disease. Adults ≥ 65 years and those with chronic conditions are eligible to receive the annual influenza vaccine free of charge. In addition, while a childhood pneumococcal conjugate vaccine (PCV) program in Mongolia reduced CAP in children, indirect benefits were not observed in adults [27, 28]. This study aimed to explore risk factors for severe CAP in adults in Ulaanbaatar, Mongolia to help guide future public health policy.

## Methods

### Study setting

In June 2016 a phased introduction of the 13-valent PCV (PCV13) into the childhood immunisation program was initiated by the government of Mongolia. PCV13 was introduced in Ulaanbaatar over three years beginning in the districts of Songinokhairkhan and Sukhbaatar before commencing in Bayanzurkh in July 2017 and the remaining six districts (including Chingeltei) in March 2018 [28]. In March 2019 an active, hospital-based surveillance for adult all-cause CAP was established in four districts of Ulaanbaatar (Songinokhairkhan, Sukhbaatar, Bayanzurkh and Chingeltei) to determine the indirect effects of childhood PCV on adult CAP [28]. Data collection extended to February 2022, with part of the surveillance system operating during the COVID-19 pandemic [29]. Mongolia began implementing COVID-19 precautions in January 2020, with increasing restrictions from February 2020 and the first locally transmitted case of COVID-19 infection detected in November 2020 [28]. Educational institutions moved online and all major public events were banned in February 2020 with Mongolia’s border being completely closed in March 2020. The use of face masks was highly encouraged and the use of hand sanitiser was made mandatory among customer services and businesses in Ulaanbaatar city [29]. COVID-19 vaccines became available in-country from February 2021.

### Study design and data collection

#### Recruitment and eligibility, inclusion and exclusion criteria

Secondary analysis was conducted using data collected from the prospective adult CAP hospital-based surveillance system between 01 March 2019 and 28 February 2022 [30]. Study participants included adults aged 18 years and older who were admitted to one of the four participating district hospitals, met the clinical CAP case definition, had resided within one of the four districts for at least three months and provided informed consent. Clinical CAP was defined as a diagnosis of pneumonia both on admission and discharge plus two or more of the following clinical signs and symptoms, at least one of which is respiratory: subjective fever/chills or documented fever (≥ 38.0 °C), new cough or change in chronic cough, new or increased sputum production, dyspnoea or tachypnoea (i.e. difficult breathing or respiratory rate > 25 breaths/minute), lung findings on auscultation (rales, decreased breath sounds), leucocytosis (> 11.0 × 10^9^ cells/l) or leukopenia (< 4.0 × 10^9^ cells/l), hypoxia (SaO_2_ < 90%), respiratory distress, or pleuritic chest pain [30, 31]. Patients who were included in the study had clinical information collected using an adapted case report form with details regarding presentation and risk factors for carriage and disease. Information regarding underlying medical conditions and CAP severity markers were collected and digital chest x-rays, nasopharyngeal swabs and urine samples were taken [30]. Any adults who had verified receipt of a pneumococcal vaccine were excluded from the study. From 2020, patients were tested for SARS-CoV-2 upon admission and those who tested positive were transferred to a specialised hospital for treatment and excluded from the study.

Recorded risk factors included previous admissions for CAP and other conditions, level of education, PCV13 vaccination status of children under 5 years living in the household, dwelling type, type of fuel used in household, tobacco use, alcohol use, fuel use related to occupation, monthly household income, comorbidities (including chronic cardiac disease, hypertension, chronic lung disease, asthma, chronic renal disease, neurological conditions, cancer, diabetes and pregnancy), pneumococcal carriage and serotype-specific urine antigen detection (SSUAD) positivity.

Severe clinical CAP was defined as cases with clinical CAP who present with two or more severity signs: presence of confusion (Glasgow coma scale < 15), hypotension (systolic blood pressure ≤ 90mmHg or diastolic blood pressure ≤ 60mmHg), tachypnoea (respiratory rate ≥ 30 breaths a minute) or hypoxaemia (oxygen saturation < 90%). Additionally all CAP patients who were admitted to the intensive care unit (ICU), received pressor support in ICU or who died were considered to have severe CAP [30, 32, 33].

#### Clinical and laboratory methodology

Clinical and laboratory methods have been previously published [30]. In brief, nasopharyngeal swabs were collected and stored according to World Health Organization recommendations [34]. Samples were shipped to the Murdoch Children’s Research Institute in Australia and screened for pneumococci using the quantitative polymerase chain reaction. Samples that were positive for pneumococci on quantitative polymerase chain reaction underwent molecular serotyping by microarray following culture amplification [35]. Urine samples were frozen at −70 °C and shipped to Pfizer laboratories in Pearl River, USA for testing using two SSUAD assays – UAD1 which detects serotypes in PCV13 and cross-reactive serotypes 6A/C, 7 F/A, 9V/9A, 18C/18A, B,F and UAD2 which detects the additional 11 serotypes in PPSV23 and cross-reactive serotypes 10A/39, 11A/11D,F, 15B/15C, 22F/22A, 33 F/33A,C, 17F/17A, 20 (20A and 20B) [36, 37]. Digital chest x-rays were re-read according to adapted standardised WHO criteria, and categorised as primary end-point pneumonia (presence of focal consolidation or pleural effusion in the lateral pleural space associated with a parenchymal infiltrate), other infiltrates (patchy interstitial infiltrates without pleural effusion or focal end-point consolidation) or negative (no consolidation, infiltrate or effusion) [30, 31]. Testing for RSV and influenza was conducted at MCRI using a validated multiplex real-time qPCR [30].

### Statistical analysis

Data was analysed using Stata version 18.0 (StataCorp LP, College Station TX). Characteristic and risk factor data were tabulated and presented using descriptive statistics and percentages. Unadjusted odds ratios (ORs) and adjusted ORs (aORs) were estimated using logistic regression models. The final multivariable logistic regression model included clinically significant risk factors for severe CAP chosen based on previous literature, applicability to respiratory infections and those with p-value < 0.05 in univariate analysis. If variables examined analogous risk factors, those with fewer missing data were chosen.

All variables were categorised as binary variables in the logistic regression models. For example, comorbidities were categorised as no and any underlying medical conditions, while chest x-ray findings were categorised as primary endpoint pneumonia and no primary endpoint pneumonia, as primary endpoint pneumonia is considered the most specific endpoint for evaluating pneumococcal aetiology.

Interactions between age and underlying medical conditions were examined. We tested for multicollinearity in the final multivariable logistic regression model using the variance inflation factor. Participants with complete data for all variables were included in the model (complete case analysis).

To explore whether characteristics of patients admitted with severe CAP changed during the COVID-19 pandemic, we compared participant characteristics between the pre-COVID-19 pandemic (March 2019 - January 2020) and during-COVID-19 pandemic (February 2020- February 2022) periods using a chi-square test.

## Results

A total of 3442 adults were admitted with CAP to the four district hospitals between March 2019 and February 2022; 3178 (92.3%) met the study case definition for clinical CAP, and of those 322 (10.1%) had severe CAP. Table 1 shows the demographic characteristics of adults hospitalised with clinical CAP. The majority of cases for both severe (125 (38.8%)) and non-severe CAP (1025 (36.8%)) were enrolled in the 46–64-year age group. The median age of participants with severe pneumonia (58.9 [IQR 46.2–71.3] years) was older than participants with non-severe pneumonia (53.7 [IQR 36.8–65.7] years, *p* < 0.001). Females accounted for a higher percentage (56.9%) of all clinical CAP admissions, while males made up over half (186 (57.8%)) of the severe CAP cases. Most participants (2724 (86.3%)) did not have any children under 5 years living in their household. Of adult patients living with children < 5 years, around half (52.4%) belonged to the 26–45-year age group.

**Table 1:** Demographic characteristics of adults hospitalised with clinical community acquired pneumonia (CAP) by pneumonia severity (*n* = 3178)^1^

Variables		Severe pneumonia *N* = 322 *n* (%)	Non-severe clinical pneumonia *N* = 2856 *n* (%)	All clinical pneumonia admissions *N* = 3178 *n* (%)
Demographics				
Age (years)	18–25	16 (5.0)	249 (8.8)	265 (8.3)
	26–45	64 (19.9)	806 (28.2)	870 (27.4)
	46–64	125 (38.8)	1052 (36.8)	1177 (37.0)
	≥ 65	117 (36.3)	749 (26.2)	866 (27.3)
	Median (IQR)	58.9 (46.2–71.3)	53.7 (36.8–65.7)	54.4 (37.9–66.0)
Gender	Male	186 (57.8)	1184 (41.5)	1370 (43.1)
	Female	136 (42.2)	1672 (58.5)	1808 (56.9)
District of admission hospital	Bayanzurkh	89 (27.6)	817 (28.6)	906 (28.5)
	Songinokhairkhan	101 (31.4)	1048 (36.7)	1149 (36.2)
	Sukhbaatar	68 (21.1)	678 (23.7)	746 (23.5)
	Chingeltei	64 (19.9)	313 (11.0)	377 (11.8)
Season	June-August (Summer)	79 (24.5)	561 (19.6)	640 (20.1)
	September-October (Autumn)	54 (16.8)	600 (21.0)	654 (20.6)
	November- February (Winter)	121 (37.6)	1181 (41.4)	1302 (41.0)
	March-May (Spring)	68 (21.1)	514 (18.0)	582 (18.3)
Risk factors				
Underlying medical conditions	No underlying medical condition	72 (22.4)	1169 (40.9)	1241 (39.0)
	Underlying medical condition	247 (76.7)	1681 (58.9)	1928 (60.7)
	Missing data	3 (0.9)	6 (0.2)	9 (0.3)
Smoking status	Non-smoker	202 (62.7)	2216 (77.6)	2418 (76.1)
	Smoker	117 (36.4)	628 (22.0)	745 (23.4)
	Missing data	3 (0.9)	12 (0.4)	15 (0.5)
Alcohol intake	Does not drink alcohol	233 (72.4)	2529 (88.6)	2762 (86.9)
	Drinks alcohol	83 (25.8)	312 (10.9)	395 (12.4)
	Missing data	6 (1.8)	15 (0.5)	21 (0.6)
Previous hospital admissions	Admitted within last year	93 (28.9)	507 (17.7)	600 (18.9)
	Not admitted within last year	217 (67.4)	2285 (80.0)	2502 (78.7)
	Missing data	12 (3.7)	64 (2.2)	76 (2.4)
Antibiotics taken in the last 48 h	Yes	56 (17.4)	618 (21.6)	674 (21.2)
	No	257 (79.8)	2214 (77.5)	2471 (77.8)
	Missing data	9 (2.8)	24 (0.9)	33 (1.0)
PCV13 status of children (under 5) in household	No children in household	279 (86.6)	2445 (85.6)	2724 (85.7)
	Child not vaccinated	27 (8.4)	260 (9.1)	287 (9.0)
	Child vaccinated	10 (3.1)	135 (4.7)	145 (4.6)
	Missing data	6 (1.9)	16 (0.6)	22 (0.7)
Socio-economic factors				
Education level completed	No higher tertiary education	235 (73.0)	1847 (64.7)	2082 (65.5)
	Higher tertiary education	81 (25.2)	994 (34.8)	1075 (33.8)
	Missing data	6 (1.8)	15 (0.5)	21 (0.7)
Dwelling type	Formal (apartment/house)	216 (67.1)	2098 (73.5)	2314 (72.8)
	Informal (ger)	104 (32.3)	748 (26.2)	852 (26.8)
	Missing data	2 (0.6)	10 (0.3)	12 (0.4)
Fuel used in home	Smoky fuel (wood/coal)	201 (62.4)	1538 (53.9)	1739 (54.7)
	Non-smoky fuel (gas/electricity)	117 (36.4)	1309 (45.8)	1426 (44.9)
	Missing data	4 (1.2)	9 (0.3)	13 (0.4)
Occupation	Inside work	19 (5.9)	325 (11.4)	344 (10.8)
	Outside work	29 (9.0)	252 (8.8)	281 (8.8)
	Self or unemployed	271 (84.2)	2267 (79.4)	2538 (79.9)
	Missing data	3 (0.9)	12 (0.4)	15 (0.5)
Fuel use related to occupation	Non-smoky fuel use	51 (15.8)	886 (31.0)	937 (29.5)
	Smoky fuel use	19 (5.9)	93 (3.3)	112 (3.5)
	Not applicable	248 (77.0)	1841 (64.5)	2089 (65.7)
	Missing data	4 (1.2)	36 (1.3)	40 (1.26)
Monthly household income^2^	≤ 61USD per person/per month	110 (34.2)	798 (27.9)	908 (28.6)
	> 61USD per person/per month	160 (49.7)	1628 (57.0)	1788 (56.3)
	Missing data	52 (16.1)	430 (15.1)	482 (15.1)
Disease severity				
Outcome	Survived	275 (85.4)	2840 (99.4)	3115 (98.0)
	Died	43 (13.3)	0 (0.0)	43 (1.4)
	Missing data	4 (1.2)	16 (0.6)	20 (0.6)
Length of hospital stay	≤ 7 days	128 (39.8)	1759 (61.6)	1887 (59.4)
	8–14 days	171 (53.1)	1076 (37.7)	1247 (39.2)
	≥ 15 days	23 (7.1)	21 (0.7)	44 (1.4)
Primary endpoint pneumonia	No primary endpoint pneumonia	96 (19.8)	1514 (53.0)	1610 (50.7)
	Primary endpoint pneumonia	127 (39.4)	595 (20.8)	722 (22.7)
	Missing data	99 (30.7)	747 (26.2)	846 (26.6)
Pneumococcal carriage and disease				
Serotype specific urinary antigen detection	Positive	58 (18.0)	195 (6.8)	253 (8.0)
	Negative	232 (72.1)	2440 (85.4)	2672 (84.0)
	Missing data	32 (9.9)	221 (7.8)	253 (8.0)
Pneumococcal carriage	Pneumococcal carriage	80 (24.9)	253 (8.8)	333 (10.5)
	No pneumococcal carriage	193 (59.9)	2233 (78.2)	2426 (76.3)
	Missing data	49 (15.2)	370 (13.0)	419 (13.2)
Viral infections				
Respiratory syncytial virus infection	Infection	1 (0.3)	17 (0.6)	18 (0.6)
	No infection	274 (85.1)	2473 (86.6)	2747 (86.4)
	Missing data	47 (14.6)	366 (12.8)	413 (13.0)
Influenza infection	Infection	12 (3.7)	101 (3.5)	113 (3.6)
	No infection	263 (81.7)	2389 (83.7)	2652 (83.4)
	Missing data	47 (14.6)	366 (12.8)	413 (13.0)
Vaccination				
Influenza vaccine received in last 12 months	Vaccinated	6 (1.9)	34 (1.2)	40 (1.3)
	Unvaccinated	305 (94.7)	2768 (96.9)	3073 (96.7)
	Missing data	11 (3.4)	54 (1.9)	65 (2.0)
Any COVID-19 vaccine received	Vaccinated	60 (18.6)	989 (34.6)	1049 (33.0)
	Unvaccinated	25 (7.8)	281 (9.8)	306 (9.6)
	Ineligible for vaccination^3^	224 (69.6)	1484 (52.0)	1708 (53.7)
	Missing data	13 (4.0)	102 (3.6)	115 (3.6)
COVID-19 period				
COVID-19 period	Pre-COVID-19 (Mar 2019-Jan 2020)	100 (31.1)	865 (30.3)	965 (30.4)
	During COVID-19 (Feb 2020- Feb 2022)	222 (68.9)	1991 (69.7)	2213 (69.6)

^1^ Demographic characteristics of hospitalised adults in Mongolia, March 2019-February 2022

^2^ 61USD is equivalent to ~ 218,900 Mongolian Tugrik

^3^ Participant hospitalised prior to the COVID-19 pandemic or before vaccinations were made available

Over three quarters (76.7%) of severe CAP patients had at least one underlying medical condition compared to 58.9% of non-severe clinical CAP cases. Of those participants with underlying medical conditions (*n* = 1928), hypertension was the most common condition in both severe (151 (61.1%)) and non-severe pneumonia (1133 (67.5%)). Most underlying medical conditions were more frequent amongst those with severe compared with non-severe pneumonia including diabetes (48 (19.5%) vs. 196 (11.7%)), heart disease (77 (31.2%) vs. 310 (18.4%)), neurological conditions (45 (18.2%) vs. 128 (7.6%)) and lung disease (91 (36.8%) vs. 453 (26.9%)). A higher percentage of those with severe CAP were smokers (36.4%) or reported alcohol consumption (25.8%) compared with those with non-severe CAP where only 22.0% smoked and 10.9% reported drinking alcohol. Just over a quarter (28.9%) of participants with severe CAP had been admitted to hospital within the last year compared to 17.7% of non-severe CAP patients.

Pneumococcal carriage (24.9%) and disease (18.0%; indicated by SSUAD positivity) were higher amongst participants with severe CAP compared with non-severe CAP, of whom 8.8% and 6.8% had pneumococcal carriage or disease, respectively (Table 1). Over the course of the hospital surveillance period 131 viral infection cases were identified, including 13 participants with severe CAP who tested positive for viral infections (1 (0.3%) respiratory syncytial virus (RSV) and 12 (3.7%) influenza). Influenza vaccination coverage was low (< 2%) across both severe and non-severe pneumonia groups, while COVID-19 vaccination coverage was lower (18.6%) in the severe pneumonia compared with the non-severe CAP group (34.6%). Additional details on participant characteristics and risk factors with multiple categories are shown in Supplementary Table 1.

### Risk factor analysis for severe CAP

#### Demographic factors and risk factors

Table 2 shows the unadjusted logistic regression results for risk factors for severe CAP. Older age was associated with higher odds for severe CAP with those aged 46–64 years having a 1.85-fold increase in the odds of severe CAP (OR:1.85; 95% confidence interval (95% CI): 1.08–3.17; *p* = 0.03) and those ≥ 65 years having a 2.43-fold increase in the odds of severe CAP (OR:2.43; 95% CI: 1.41–4.18; *p* < 0.001) compared to 18–25 year olds. Additionally, males had an almost two-fold increase in the odds of severe CAP (OR:1.93; 95% CI: 1.53–2.44; *p* < 0.001) compared with females. Smoking tobacco was associated with a 2.04-fold increase (OR:2.04; 95% CI: 1.60–2.61; *p* < 0.001) and alcohol with an almost 3-fold increase in the odds of severe CAP (OR:2.89; 95% CI: 2.19–3.81; *p* < 0.001). Antibiotics taken in the last 48 h was not statistically significant in the unadjusted logistic regression results (OR:0.78; 95% CI: 0.58–1.06; *p* = 0.11).

**Table 2:** Unadjusted univariate logistic regression of risk factors for severe community acquired pneumonia (CAP) amongst hospitalised adults in Mongolia^1^

			Unadjusted logistic regression	
Variable		Severe pneumonia *N* = 322 n (%)	Odds ratio (95% confidence interval)	*P *value
Demographics				
Age (years)	18–25	16 (5.0)	Reference	
	26–45	64 (19.9)	1.24 (0.70–2.18)	0.47
	46–64	125 (38.8)	1.85 (1.08–3.17)	0.03
	≥ 65	117 (36.3)	2.43 (1.41–4.18)	0.001
Gender	Female	136 (42.2)	Reference	
	Male	186 (57.8)	1.93 (1.53–2.44)	< 0.001
District of admission hospital	Sukhbaatar	68 (21.1)	Reference	
	Songinokhairkhan	101 (31.4)	0.96 (0.70–1.53)	0.81
	Bayanzurkh	89 (27.6)	1.09 (0.78–1.51)	0.63
	Chingeltei	64 (19.9)	2.04 (1.41–2.94)	< 0.001
Risk factors				
Underlying medical conditions	No underlying medical condition	72 (22.6)	Reference	
	Underlying medical condition	247 (77.4)	2.38 (1.81–3.13)	< 0.001
Smoking	Non-smoker	202 (63.3)	Reference	
	Smoker	117 (36.7)	2.04 (1.60–2.61)	< 0.001
Alcohol intake	Does not drink alcohol	233 (73.7)	Reference	
	Drinks alcohol	83 (26.3)	2.89 (2.19–3.81)	< 0.001
Any previous hospital admissions in last year	No	229 (71.1)	Reference	
	Yes	93 (28.9)	1.88 (1.45–2.44)	< 0.001
Antibiotics taken in last 48 h	No	257 (82.1)	Reference	
	Yes	56 (17.9)	0.78 (0.58–1.06)	0.11
Socio-economic factors				
Education level	Higher tertiary education	81 (25.6)	Reference	
	No higher tertiary education	235 (74.4)	1.56 (1.20–2.03)	0.001
Dwelling type	Formal (apartment/house)	216 (67.5)	Reference	
	Informal (ger)	104 (32.5)	1.35 (1.05–1.73)	0.02
Fuel used in home	Non-smoky fuel (gas/electricity)	117 (36.8)	Reference	
	Smoky fuel (wood/coal)	201 (63.2)	1.46 (1.15–1.86)	< 0.001
Fuel use related to occupation	Non-smoky fuel use	51 (16.0)	Reference	
	Smoky fuel use	19 (6.0)	3.55 (2.01–6.27)	< 0.001
	Not applicable	248 (78.0)	2.32 (1.70–3.17)	< 0.001
Monthly household income^2^	> 61USD per person/per month	160 (59.3)	Reference	
	≤ 61USD per person/per month	110 (40.7)	1.40 (1.08–1.81)	0.01
Pneumococcal carriage and disease				
Serotype specific urinary antigen detection	Negative	232 (80.0)	Reference	
	Positive	58 (20.0)	3.13 (2.66–4.32)	< 0.001
All pneumococcal carriage	No pneumococcal carriage	193 (70.7)	Reference	
	All pneumococcal carriage	80 (29.3)	3.66 (2.73–4.89)	< 0.001
COVID-19 period				
COVID-19 period	Pre-COVID-19 (Mar 2019-Jan 2020)	100 (31.1)	Reference	
	During COVID-19 (Feb 2020- Feb 2022)	222 (68.9)	0.96 (0.75–1.24)	0.78

^1^ Data was collected from hospitalised patients between March 2019-February 2022

^2^ 61USD is equivalent to ~ 218,900 Mongolian Tugrik

On multivariable logistic regression analysis (Table 3) any alcohol consumption was associated with a > 2-fold increase in the odds of severe CAP (aOR:2.19; 95% CI:1.49–3.22; *p* < 0.001), and smoking was associated with an increased odds of severe CAP (aOR:1.53; 95% CI:1.08–2.16; *p* = 0.01). Previous hospital admission within the last year (aOR:1.73; 95% CI:1.26–2.37; *p* = 0.001), and the presence of an underlying medical condition (aOR:1.96; 95% CI:1.39–2.76; *p* < 0.001) were also associated with an increase in the odds of severe CAP. Age was no longer significant at the 95% confidence interval, although the point estimates still suggested age was a risk factor. Interactions between age and underlying medical conditions were non-significant.

**Table 3:** Multivariable logistic regression of risk factors for severe community acquired pneumonia (CAP) amongst hospitalised adults in Mongolia

			Adjusted logistic regression	
Variable		Severe pneumonia *N* = 322 n (%)	Odds ratio (95% confidence interval)	*P* value
Demographics				
Age (years)	18–25	16 (5.0)	Reference	
	26–45	64 (19.9)	0.94 (0.48–1.83)	0.86
	46–64	125 (38.8)	1.03 (0.54–1.97)	0.93
	≥ 65	117 (36.3)	1.37 (0.71–2.63)	0.35
Risk factors				
Underlying medical conditions	No underlying medical condition	72 (22.6)	Reference	
	Underlying medical condition	247 (77.4)	1.96 (1.39–2.76)	< 0.001
Smoking	Non-smoker	202 (63.3)	Reference	
	Smoker	117 (36.7)	1.53 (1.08–2.16)	0.01
Alcohol intake	Does not drink alcohol	233 (73.7)	Reference	
	Drinks alcohol	83 (26.3)	2.19 (1.49–3.22)	< 0.001
Any previous hospital admissions in last year	No	229 (71.1)	Reference	
	Yes	93 (28.9)	1.73 (1.26–2.37)	0.001
Socio-economic factors				
Education level	Higher tertiary education	81 (25.6)	Reference	
	No higher tertiary education	235 (74.4)	1.13 (0.82–1.56)	0.44
Fuel used in home	Non-smoky fuel (gas/electricity)	117 (36.8)	Reference	
	Smoky fuel (wood/coal)	201 (63.2)	1.17 (0.88–1.58)	0.28
Pneumococcal carriage and disease				
Serotype specific urinary antigen detection	Negative	232 (80.0)	Reference	
	Positive	58 (20.0)	1.69 (1.11–2.57)	0.01
All pneumococcal carriage	No pneumococcal carriage	193 (70.7)	Reference	
	All pneumococcal carriage	80 (29.3)	2.67 (1.88–3.79)	< 0.001

Data was collected from hospitalised patients between March 2019-February 2022

There was limited collinearity between variables with a mean model VIF = 1.17 (continuous age)

#### Socioeconomic factors

A number of socioeconomic risk factors were associated with increased odds of severe CAP in the unadjusted logistic regression analysis. Not having tertiary education was associated with 1.56-fold increased odds (OR:1.56; 95% CI: 1.20–2.03; *p* = 0.001), Living in an informal (ger) dwelling was associated with 1.35-fold increased odds (OR:1.35; 95% CI: 1.05–1.73; *p* = 0.02), using a smoky fuel in the house associated with 1.46 increased odds (OR:1.46; 95% CI: 1.15–1.86; *p* < 0.001), exposure to a smoky fuel at work a 3.55-fold increase (OR:3.55; 95% CI: 2.01–6.27; *p* < 0.001) and a monthly household income per person of equal to or below the poverty line was associated with a 1.40-fold increase (OR:1.40; 95% CI: 1.08–1.81; *p* = 0.01) in the odds of severe CAP. On adjusted multivariable analysis, education level (aOR:1.13; 95% CI:0.82–1.56; *p* = 0.44) and fuel used in the home (aOR:1.17; 95% CI:0.88–1.58; *p* = 0.28) were no longer associated with an increased odds of severe CAP.

#### Pneumococcal and disease carriage factors

Both pneumococcal carriage and SSUAD positivity were associated with a greater than three-fold increase in the odds of severe CAP with an unadjusted odds ratio of 3.66 (OR:3.66; 95% CI: 2.73–4.89; *p* < 0.001) and 3.13 (OR:3.13; 95% CI: 2.66–4.32; *p* < 0.001) respectively on univariate analysis.

On multivariable analysis, any pneumococcal carriage was associated with an almost three-fold increase in the odds of severe CAP (aOR:2.67; 95% CI:1.88–3.79; *p* < 0.001), while SSUAD positivity was associated with a 1.69-fold increase in the odds of severe CAP (aOR:1.69; 95% CI:1.11–2.57; *p* = 0.01).

### Impact of COVID-19 pandemic on study population

When stratified by the pre- and during COVID-19 pandemic periods, and evaluating the same risk factors, differences between periods were limited to pneumococcal carriage, SSUAD positivity and viral infection cases (Table 4). A higher percentage of severe CAP patients had pneumococcal disease in the pre-COVID-19 period (28.0%) compared to the COVID-19 period (16.2%, *p* = 0.02). Similarly, 39.6% of severe CAP patients were pneumococcal carriers in the pre-pandemic period, whilst only 24.2% were carriers during the pandemic period (*p* = 0.008). In the pre-COVID-19 period 12% of patients with severe CAP had a viral infection with either RSV or influenza whereas only 1% had a viral infection during the COVID-19 period (*p* < 0.001).

**Table 4:** Comparison of characteristics of severe community acquired pneumonia (CAP) patients in Mongolia pre- and during the COVID-19 pandemic

Variables		Pre-COVID-19 pandemic period *n* = 100 *n* (%)	During-COVID-19 pandemic period *n* = 222 *n* (%)	*P* value
Age (years)	18–25	7 (7.0)	9 (4.1)	0.60
	26–45	20 (20.0)	44 (19.8)	
	46–64	35 (35.0)	90 (40.5)	
	≥ 65	38 (38)	79 (35.6)	
Gender	Male	57 (57.0)	129 (58.1)	0.85
	Female	43 (43.0)	93 (41.9)	
District	Bayanzurkh	25 (25.0)	64 (28.8)	0.17
	Songinokhairkhan	26 (26.0)	75 (33.8)	
	Sukhbaatar	28 (28.0)	40 (18.0)	
	Chingeltei	21 (21.0)	43 (19.4)	
Underlying medical conditions	No underlying medical condition	25 (26.3)	47 (22.4)	0.44
	Underlying medical condition	74 (74.7)	173 (78.6)	
Dwelling type	Formal (apartment/house)	67 (67.0)	149 (67.7)	0.90
	Informal (ger)	33 (33.0)	71 (32.3)	
Outcome	Survived	88 (89.8)	187 (85.0)	0.25
	Died	10 (10.2)	33 (15.0)	
Length of stay in hospital	1–7 days	37 (37.0)	91 (41.0)	0.50
	> 7 days	63 (63.0)	131 (59.0)	
Any previous admissions	Yes	53 (54.6)	105 (49.3)	0.38
	No	44 (45.4)	108 (50.7)	
Previous antibiotics in last 48 h	Yes	17 (17.7)	39 (18.0)	0.96
	No	79 (82.3)	178 (82.0)	
Primary endpoint pneumonia	No primary endpoint pneumonia	24 (40.7)	72 (43.9)	0.67
	Primary endpoint pneumonia	35 (59.3)	92 (56.1)	
Serotype specific urinary antigen detection	Positive	26 (28.0)	32 (16.2)	0.02
	Negative	67 (72.0)	165 (83.8)	
All pneumococcal carriage	All pneumococcal carriage	36 (39.6)	44 (24.2)	0.008
	No pneumococcal carriage	55 (60.4)	138 (75.8)	
Viral infection (Influenza or RSV)	Infection	11 (12.0)	2 (1.1)	< 0.001
	No infection	81 (88.0)	181 (98.9)	

Pre-COVID-19 pandemic period was considered between March 2019 and January 2020 and the during-COVID-19 pandemic period was defined as February 2020 to February 2022

## Discussion

This study describes risk factors for severe CAP in adults aged ≥ 18 years in Ulaanbaatar, Mongolia hospitalised for pneumonia. We found that underlying medical conditions, a recent previous hospital admission, and smoking or alcohol use increased the likelihood of hospitalisation with severe CAP. In addition, pneumococcal disease or carriage was also associated with severe CAP. This study helps address a regional data gap and will assist in designing targeted CAP prevention strategies for adults.

*S. pneumoniae* remains the leading cause of adult CAP globally, with the pneumococcus being the most frequently found infection in severe CAP cases [2, 20, 38]. Our analysis found that vaccine serotype specific pneumococcal disease (identified by SSUAD) or pneumococcal carriage (of any serotype) were risk factors for severe CAP within the population. In adults pneumococcal pneumonia is associated with intensive care admissions, high mortality and higher severity scores e.g. CURB-65 score (confusion, uraemia, raised respiratory rate, low blood pressure, age ≥ 65 years) compared to pneumococcal negative CAP [18, 20, 21, 39, 40]. PCVs have been shown to be a protective factor against the onset of CAP, reducing severity and risk of mortality [41, 42]. The frequencies of viral infections such as influenza and RSV were low during the study period likely due to mitigation strategies implemented to prevent the spread of COVID-19. The low number of viral infections limited our ability to use them in the analysis. However these viruses have been shown to be leading causative agents of acute respiratory infections including CAP in adults, especially those that are older [4, 5, 43]. Vaccinations against these viruses have been shown to protect older adults and those with underlying medical conditions, with a reduced burden of disease and less severe onset of CAP symptoms [44–46]. Although annual influenza is recommended for high-risk adults in Mongolia, there are currently no recommendations or availability of pneumococcal vaccination for adults in Mongolia, with PCV13 only accessible as part of the childhood immunisation program.

Older age is frequently observed as a risk factor for CAP in adults, with those aged ≥ 65 years experiencing a higher incidence of CAP, higher rates of hospitalisations, complications and mortality [7, 13, 47, 48]. Studies from India and Japan identified older age (≥ 60 and ≥ 65 respectively) as risk factors for severe CAP [38, 49]. In our multivariable analysis, age was no longer significant at the 95% confidence interval, although the point estimates still suggested age was a risk factor, with the highest estimates being observed for those aged ≥ 65 years. The robustness of association between older age and severe CAP may have been impacted by the confounding effect between age and underlying medical conditions. Whilst this analysis did not identify age as a significant risk factor for severe CAP at the 95% confidence interval, the older age group still experienced the highest proportions of deaths, longest hospital stays and highest number of ICU admissions. A retrospective analysis which described the epidemiology of hospitalised adults with CAP in Mongolia, found that individuals in the > 65-year age group made up the largest proportion of severe CAP cases and had an almost seven-fold increase in the odds of severe CAP on univariable analysis [25].

We found that underlying medical conditions were associated with an increased risk of severe CAP. This is consistent with research from HICs and literature reviews that identified that individuals with conditions such as chronic obstructive pulmonary disease, chronic heart disease and diabetes were more likely to experience severe CAP [15, 38, 47]. These comorbidities predispose individuals to CAP, more severe illness and a greater chance of more adverse outcomes [47]. Whilst our analysis looked at all underlying medical conditions combined to increase the precision of estimates, results nevertheless highlighted the role of underlying medical conditions in increasing the odds of severe CAP in Mongolian adults.

Smoking tobacco cigarettes was identified as a risk factor for severe CAP in this population. Cigarette smoking is an acknowledged risk factor for CAP and increased risk of mortality, with more frequent smoking being associated with a higher risk of CAP [12, 50, 51]. There is a strong link between those who smoke and bacterial infections increasing the likelihood of pneumococcal pneumonia and mortality in this group [50, 51].

Alcohol consumption, another potentially modifiable factor, was also identified as a risk factor for severe CAP. Only 26% of patients with severe CAP indicated that they drank alcohol and over half of those identified as light-moderate drinkers. Previous studies have shown mixed results for alcohol consumption as a risk factor for CAP and severe CAP, with alcohol overconsumption or heavy alcohol drinking showing the strongest relationship [9, 11]. A strong association and co-occurrence between smoking and drinking may point to alcohol consumption as a confounder rather than a risk factor [52, 53].

Recent previous hospital admissions were identified as a risk factor for severe CAP in adults in Mongolia. Other studies have found that previous hospital admissions for CAP and other respiratory infections are risk factors for CAP in adults [8]. For our analysis we combined previous CAP and any other admissions due to small numbers. With the possible reasons for non-pneumonia related prior admissions differing, the implications related to the possible risk attached to these admissions may be variable and may be related to underlying medical conditions driving some of these admissions.

In this analysis we identified key risk factors for severe CAP in hospitalised adults in urban Mongolia which can be used to prioritise high risk groups for possible interventions. These interventions include a continuation of public health campaigns to reduce excessive alcohol consumption and tobacco use amongst the population. With an estimated 50% of males in Mongolia who smoke this is a modifiable risk factor to reduce CAP burden amongst males who were represented at a higher rate amongst severe CAP patients. Increasing access to vaccination amongst high risk populations would also play a role in reducing the burden of CAP. Whilst this analysis only included pneumococcal carriage and disease as risk factors for severe CAP, viruses such as influenza and RSV have been shown to increase the burden of CAP [44–46]. Vaccinations against the pneumococcus, influenza, RSV and COVID-19 have been shown to prevent CAP, reduce severity of disease and risk of mortality especially in the elderly [41, 42, 44–46, 54]. Although limited to a subset of participants, COVID-19 vaccination coverage was lower in those with severe compared to non-severe pneumonia. The US CDC recommends influenza vaccine for all adults, COVID-19 vaccine for adults aged ≥ 65 years and those at high risk for severe COVID-19, the RSV vaccine for all adults aged ≥ 75 years and those 60–74 years with risk factors for severe disease. They recently lowered their age-based recommendation for adult PCV from 65 years old to 50 years old due to a high disease burden [55]. In Mongolia and other LMICs, adult vaccination programs will likely need to be more targeted and aimed at high-risk groups, including those with underlying medical condition or 65 years and older. In addition, the cost of RSV vaccines may also limit use in these settings with the vaccine currently inaccessible in Mongolia. Given that prior hospitalisation was a risk factor for severe CAP in our study offering adults, such as those 50 years and older with underlying medical conditions, PCV on discharge may be another opportunity for targeted interventions.

Our analysis has a number of strengths. It has expanded on the limited evidence base surrounding risk factors for severe CAP in adults in LMICs. Our data provides context for risk factors for severe CAP in LMICs with a similar climate, socioeconomic setting or high level of air pollution. Secondly our analysis was nested within a larger research project with well-established methods and trained staff. The prospective adult surveillance used standard operating procedures and structured questionnaires leading to high comparability and standardisation between the various hospital locations. However, some of the data was self-reported including alcohol consumption and frequency of smoking which may have led to underreporting of these variables. Other limitations of this research include the limited detection of viral co-infections with RSV and influenza within the population, impacting the ability to examine the risks associated with viral co-infection and severe CAP. A proportion of the study recruitment was during the COVID-19 pandemic period and implementation of stringent public health measure likely reduced viral infections. We compared participant characteristics between the pre- and during COVID-19 period and the only differences observed were a decrease in viral infections, pneumococcal carriage and pneumococcal disease in the COVID-19 period. We were not able to collect data on all risk factors for severe CAP in this research, including outdoor air pollution exposure which would be especially relevant due to the poor air quality in Ulaanbaatar.

## Conclusion

We identified key risk factors for severe CAP in hospitalised adults in urban Mongolia. Our findings suggest that the implementation of a targeted adult-based pneumococcal vaccine program for older adults and all adults with co-morbidities could be a valuable approach in reducing cases of severe CAP. In addition, there should be continued implementation of additional health policies and programs aimed at decreasing cigarette smoking and excessive alcohol use.

## Supplementary Information

Supplementary Material 1: Supplementary Table 1. Describes demographic characteristics and risk factors with multiple categories in adults hospitalised with clinical community acquired pneumonia by severity status. The data in this table expands upon the binary categories used for these variables in Table 1.

## Data Availability

All data generated or analysed during this study are included in this published article.

## Abbreviations

aOR: adjusted odds ratio
CAP: Community acquired pneumonia
COVID-19: Coronavirus disease 2019
HICs: High-income countries
ICU: Intensive care unit
LMICs: Low-middle income countries
PCV: Pneumococcal conjugate vaccine
PCV13: 13-valent PCV
SSUAD: Serotype-specific urine antigen detection
95% CI: 95% confidence interval
