# Supplementary material for: Factors associated with severe pneumonia in adults hospitalised with community-acquired pneumonia in Mongolia
Source: BMC Public Health. 2026 Jan 3;26:431. doi: 10.1186/s12889-025-26109-2 (PMC12865962; doi:10.1186/s12889-025-26109-2)
Supplement: Supplementary file 1 — Supplementary Material 1: Supplementary Table 1. Describes demographic characteristics and risk factors with multiple categories in adults hospitalised with clinical community acquired pneumonia by severity status. The data in this table expands upon the binary categories used for these variables in Table 1. [file 12889_2025_26109_MOESM1_ESM.pdf]

Supplementary information

Supplementary Table 1 describes demographic characteristics and risk factors with multiple categories in adults hospitalised with clinical community acquired pneumonia by severity status. The data in this table expands upon the binary categories used for these variables in Table 1.

Supplementary Table 1*: Expanded risk factors and education level characteristics of adults hospitalised with clinical community acquired pneumonia (CAP) by severity status.*

Variables		Severe pneumonia N=322 n (%)	Non-severe clinical pneumonia N=2856 n (%)	All clinical pneumonia admissions N=3178 n (%)
**Risk factors**				
Underlying medical conditions	No underlying medical condition	72 (22.4)	1169 (40.9)	1241 (39.0)
	One underlying medical condition	81 (25.1)	899 (31.5)	980 (30.8)
	More than one underlying medical conditions	152 (47.2)	736 (25.8)	888 (28.0)
	Missing data	17 (5.3)	52 (1.8)	69 (2.2)
Smoking status	Non-smoker	202 (63.3)	2216 (77.9)	2418 (76.4)
	Light smoker (1-10 cig/day)	67 (21.0)	460 (16.2)	527 (16.7)
	Moderate smoker (11-20 cig/day)	35 (11.0)	136 (4.8)	171 (5.4)
	Heavy smoker (>20 cig/day)	4 (1.3)	7 (0.2)	11 (0.4)
	Unknown frequency of smoking	11 (3.4)	25 (0.9)	36 (1.1)
Alcohol intake	Does not drink alcohol	233 (72.4)	2530 (88.6)	2763 (86.9)
	Light-Moderate drinker (1-14 units of alcohol a week)	44 (13.7)	211 (7.4)	255 (8.0)
	Heavy drinker (>14 units of alcohol a week)	4 (1.2)	11 (0.4)	15 (0.5)
	Alcohol consumption frequency unknown[1]	35 (10.9)	90 (3.1)	125 (4.0)
	Missing data	6 (1.8)	14 (0.5)	20 (0.6)
	Within last year	93 (28.9)	462 (16.2)	549 (17.3)

Previous hospital admissions	Within one-two years	22 (6.8)	191 (6.7)	213 (6.7)
	More than two years ago	42 (13.1)	279 (9.8)	321 (10.1)
	No previous admissions	152 (47.2)	1837 (64.3)	1989 (62.6)
	Missing data	12 (3.7)	87 (3.0)	106 (3.3)
**Socio-economic factors**				
Education level completed	None	12 (3.7)	71 (2.5)	83 (2.6)
	Primary school	35 (10.9)	143 (5.0)	178 (5.6)
	High school	188 (58.4)	1633 (57.2)	1821 (57.3)
	Tertiary education	81 (25.2)	994 (34.8)	1075 (33.8)
	Missing data	6 (1.8)	15 (0.5)	21 (0.7)

[1] Participants who indicated on participant questionnaire that they drink alcohol but did not specify the frequency of alcohol consumption.
